# Predictive value of NT-pro BNP on outcomes of children with ventricular septal defect surgery

**DOI:** 10.3389/fcvm.2024.1454371

**Published:** 2025-01-15

**Authors:** Weidan Chen, Yajie Tang, Ye Lu, Li Ma, Xinxin Chen, Techang Liu

**Affiliations:** ^1^ Cardiovascular Center, Guangzhou Women and Children’s Medical Center, Guangzhou Medical University, Guangzhou, China; ^2^ Department of Cardiovascular Surgery, Fuwai Hospital, National Center for Cardiovascular Diseases, Chinese Academy of Medical Sciences and Peking Union Medical College, Beijing, China

**Keywords:** ventricular septal defect, children, NT-pro BNPNT-proBNP, predictive value, clinical outcomes

## Abstract

**Background:**

Limited study has shown whether NT-proBNP is related to the prognosis of children wth ventricular septal defect (VSD) surgery. The study was conducted to determine the predictive value of NT-proBNP on outcomes of children with VSD surgery.

**Methods:**

A total of 798 children with VSD surgery were enrolled, with NT-proBNP measured at preoperatively and 24-h postoperatively. The short- and mid-term clinical outcomes were recorded. Propensity scores (PS) was performed to acquire pre-op and post NT-proBNP 24-h PS-matched cohorts for comparisons between groups.

**Results:**

In the pre NT-proBNP PS-matched cohort, the higher NT pro-BNP group had longer hospitalization time and lower post-op 1-month EF value compared with low NT pro-BNP group (all *P* < 0.05), and there wasn't significant difference of mechanical ventilation time, cardiopulmonary bypass (CPB) time, intensive care unit (CCU) stay, and ejection fraction (EF) values of 3 month to 12 months after surgery (all *P* > 0.05). In the post NT-proBNP PS-matched cohort, there wasn't significant difference of mechanical ventilation time, CPB time, CCU stay, hospitalization time, and EF values of 1 month to 12 months after surgery between two groups (all *P* > 0.05).

**Conclusions:**

VSD children with higher pre NT-proBNP level had longer hospital stays after surgery than those with lower level. Pre NT-proBNP level had no effect on mechanical ventilation time, CPB time, ACC time and CCU stay and cardiac function after 3 months postoperatively. Post-op 24-h NT pro-BNP level wasn't associated with clinical outcomes.

## Introduction

Ventricular septal defect (VSD) is one of the most common congenital cardiac structural anomalies. While a majority can close spontaneously, a proportion still requires intervention through surgical or percutaneous approaches (1, 2). Hemodynamic abnormalities in VSD can lead to activation of cardiomyocytes and secretion of neurohormones, resulting in cardiac function alteration (3, 4). Brain natriuretic peptide (BNP) is a cardiac neurohormone, mainly produced by ventricular myocytes (5). When ventricular pressure or volume load increases, the endoprotease furin is activated and then cleaves BNP precursors into active c-terminal BNP 32 and N-terminal pro-B type natriuretic peptide (NT-proBNP). The c-terminal BNP 32 and NT-proBNP then alter intracellular signaling messengers and induce a series of physiological activities, including diuresis, vasodilation, and inhibition of myocardial remodeling (6, 7).

Existing studies have shown that plasma BNP level is elevated in adult patients with congestive heart failure (8). BNP level is correlated with the severity of heart failure and can predict morbidity and mortality (9). Plasma BNP has also been shown to be a useful biomarker for various other diseases, such as congenital heart disease, myocardial infarction, and cardiomyopathy in adults (8, 10, 11). Compared to the numerous studies of BNP in adult heart disease, data from children heart disease are limited. Few studies have reported the correlation between BNP and the postoperative prognosis of children with simple congenital heart disease, and studies specifically focusing on the correlation between BNP and the prognosis after VSD closure are even rarer.

We conducted this study with the hypothesis that NT-proBNP has a prognostic value in children with VSD surgery. We enrolled 798 children who underwent VSD surgery, with NT-proBNP measured preoperatively and 24-h postoperatively, with perioperative details recorded to evaluate the short-term clinical outcomes, with ejection fraction (EF) values measured by echocardiography 1-month, 3-month, 6-month and 12-month postoperatively to evaluate the mid-term clinical outcomes. All these data were analyzed together to determine whether NT-proBNP has a prognostic role in children with VSD surgery.

## Materials and methods

### Study population

Children aged <4 years who were scheduled for VSD surgical repair from January 2014 through December 2017 were eligible. Patients with structural heart lesions other than VSD, atrial septal defect (ASD), patent foramen ovale (PFO), and patent ductus arteriosus (PDA) were excluded. Finally, a total of 798 patients satisfied the inclusion criteria and were enrolled in the present study. All the patients' parents gave written informed consents to participate in the present study. The study was approved by the Research Ethics Committee of our institute.

### Blood assay for laboratory biomarkers and NT-proBNP

Laboratory biomarkers on admission were tested and recorded, including white blood cells (WBC), hemoglobin (Hb), creatinine, albumin (Alb), Na^+^, K^+^, total bilirubin (TBIL), direct bilirubin (DBIL), alanine aminotransferase (ALT), aspartate amino transferase (AST). In addition, plasma NT-proBNP levels were measured preoperatively and 24-h postoperatively.

### Collection and records for clinical data

All patients' clinical data, both basic characteristics and perioperative details, including mechanical ventilation time, cardiopulmonary bypass (CPB) time, aorta cross-clamp (ACC) time, cardiac care unit (CCU) stay, and hospitalization time were collected and recorded.

### Echocardiographic evaluation

Ultrasound examinations of the VSD children were performed using a Philips ultrasound machine (EPIQ7; Philips Medical Systems, USA) with a 2–5 MHz cardiac transducer. All ultrasound examinations were performed by qualified doctors who were blinded to the results of the NT-proBNP measurements, and each doctor had more than 10 years of experience in the field of echocardiography. Detailed anatomical and functional examinations were performed for all children. A standard VSD measurement was performed, and EF was used as a vital marker of cardiac function. Echocardiographic evaluation was performed preoperatively, 24-h, 1-month, 3-month, 6- month and 12-month postoperatively.

### Clinical outcomes

The short-term clinical outcome was evaluated by perioperative conditions, including the mechanical ventilation time, CPB time, ACC time, CCU stay, and hospitalization time.

The mid-term clinical outcome was evaluated by EF values in 1 month, 3 months, 6 months, and 12 months after surgery.

### Groups and cohorts

Based on the preoperative (pre-op) and postoperative (post-op) 24-h NT-proBNP level, patients were divided into high and low NT pro-BNP groups, respectively. High NT pro-BNP level was defined as ≥75th percentile and low NT pro-BNP level was defined as <25th percentile.

The overall cohort was composed of 798 VSD patients. Based on pre-op NT-proBNP level, patients in the high and low NT-proBNP groups were performed by propensity score (PS) matching for baseline characteristics to obtain the pre NT-proBNP PS-matched cohort, making clinical outcomes comparable between the two groups. Based on post-op NT-proBNP level, the same operation was performed to obtain post NT-proBNP PS-matched cohort.

### Statistical analysis

Normally distributed continuous variables are displayed as the mean ± standard deviation and differences between two groups were compared using the Student's *t*-test. Continuous variables with skewed distributions are expressed as the median (25th–75th percentile), and comparisons between groups were conducted using the Wilcoxon rank-sum test or Kruskal-Wallis test. Descriptive analysis of count data was performed by frequency and constituent ratio, and comparison of differences between groups was performed by chi-square test or Kruskal-Wallis test. Correlation between BNP and clinical outcome was determined by Spearman test. PS included covariates that may affect both the short-term and mid-term outcomes of patients, and that were unbalanced between low and high NT pro-BNP groups. PS were calculated within the whole cohort using multivariable logistic regression models (12, 13). All the statistical tests were 2-sided and *P*-values <0.05 were considered statistically significant. Statistical analyses were performed using IBM SPSS 22.0 statistical software (SPSS Inc., Chicago, IL, USA) and R (version 3.6.0; R Development Core Team) within the RStudio (version 1.1.463) platform.

## Results

1. Description of NT pro-BNP in the overall cohort

**Figure 1:**
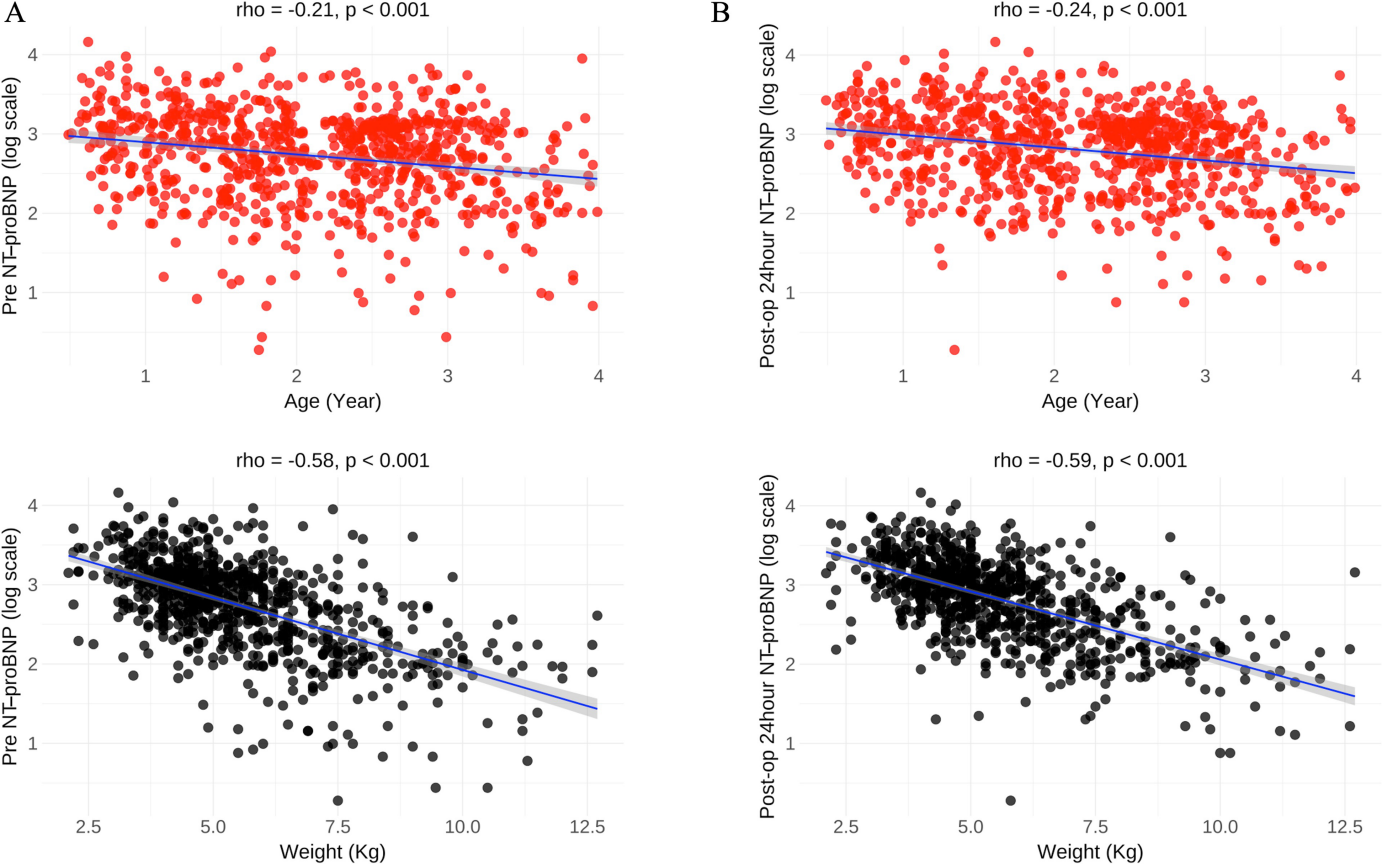
Correlations between NT pro-BNP and age, weight in the overall cohort. **(A)** Pre NT-proBNP level was negatively correlated with age and weight; **(B)** Post-op 24-h NT pro-BNP level was negatively correlated with age and weight. Pre-op, preoperative. Post-op, postoperative.

A total of 798 patients (mean age 2.1 year) who underwent VSD surgery were included in the study. In the overall cohort, pre-op and post-op 24-h NT pro-BNP levels were 1,107.5 (352.1–2,453.2) pg/ml and 1,405.8 (496.8–2,700.8) pg/ml. Post-op 24-h NT pro-BNP was significantly increased compared with the pre-op one (z = 2.82, *P* = 0.005). Median pre NT-proBNP levels in male and female were 1,100.2 and 1,117.0 pg/ml (median change of 16.8, *P* = 0.82), and median post-op 24-h NT pro-BNP levels in male and female were 1,361.8 and 1,441.9 pg/ml (median change of 80.1, *P* = 0.48). Pre-op and post-op 24-h NT pro-BNP levels were negatively correlated with age (r=−0.21, *p* < 0.001; r=−0.24, *p* < 0.001;) and weight (r=−0.58, *p* < 0.001; r = −0.59, *p* < 0.001) (Figure 1).

(1) Baseline characteristics of VSD patients between high and low NT pro-BNP groups

In the overall cohort, a total of 599 patients were in low NT pro-BNP group and 199 patients in high NT pro-BNP group. High NT pro-BNP level was calculated as 2,453.2 pg/ml (pre-op) and 2,700.8 pg/ml (post-op 24-h). The results of demographics, comorbidities and complications, laboratory on admission of patients in the low and high NT pro-BNP groups are summarized in Table 1. Compared to low NT pro-BNP group, patients in the high NT pro-BNP group were younger and weighed less, had higher rates of pulmonitis (all *P* < 0.05). In addition, there were significant differences of creatinine, Alb, Na^+^, TBIL, DBIL between the two groups (all *P* < 0.05).

**Table 1:** Patient characteristics of low and high NT pro-BNP groups in overall cohort.

	All	Pre NT-proBNP			Post- 24 h NT pro-BNP		
		Low	High	*P*	Low	High	*P*
Demographics							
Age (year, Mean ± SD)	2.1 ± 0.8	2.2 ± 0.8	1.9 ± 0.8	<0.001	2.2 ± 0.8	1.8 ± 0.8	<0.001
Gender (%)							
Male	479 (60.0%)	354 (59.1%)	125 (62.8%)	0.354	354 (59.1%)	125 (62.8%)	0.354
Female	319 (40.0%)	245 (40.9%)	74 (37.2%)		245 (40.9%)	74 (37.2%)	
Weight (kg, Mean ± SD)	5.6 ± 1.9	6.03 ± 1.93	4.44 ± 1.17	<0.001	6.03 ± 1.94	4.44 ± 1.12	<0.001
Comorbidities and complications							
Pulmonitis (%)	108 (13.5)	55 (9.2%)	53 (26.6%)	<0.001	70 (11.7%)	38 (19.1%)	<0.010
Trisomy 21 syndrome (%)	15 (1.6)	9 (1.5%)	6 (3.0%)	0.228	10 (1.7%)	5 (2.5%)	0.450
Laboratory on admission							
WBC (mcL, Mean ± SD)	10.1 ± 4.2	10.2 ± 4.2	9.6 ± 4.5	0.050	10.3 ± 4.1	9.5 ± 4.7	0.021
Hemoglobin (g/dl,Mean ± SD)	104.9 ± 13.4	105.6 ± 13.0	103.0 ± 14.5	0.026	105.3 ± 13.3	103.8 ± 13.7	0.153
Creatinine (mg/dl, Mean ± SD)	21.3 ± 6.2	20.4 ± 5.2	24.0 ± 7.8	<0.001	20.7 ± 5.4	23.1 ± 7.8	<0.001
Alb (g/L, Mean ± SD)	41.5 ± 4.0	42.1 ± 3.8	39.6 ± 4.1	<0.001	42.1 ± 3.9	39.8 ± 3.9	<0.001
Na (mmol/L, Mean ± SD)	137.3 ± 2.8	137.5 ± 2.6	136.7 ± 3.1	<0.001	137.4 ± 2.7	136.9 ± 2.9	0.026
K (mmol/L, Mean ± SD)	4.3 ± 0.6	4.3 ± 0.6	4.3 ± 0.7	0.680	4.3 ± 0.5	4.3 ± 0.7	0.240
TBIL (mg/dl, Mean ± SD)	15.7 ± 26.9	11.7 ± 17.9	27.7 ± 41.9	<0.001	12.0 ± 20.5	26.8 ± 38.6	<0.001
DBIL (mg/dl, Mean ± SD)	4.7 ± 4.7	3.9 ± 3.8	7.1 ± 6.3	<0.001	3.9 ± 3.9	7.1 ± 5.9	<0.001
AST (U/L, Mean ± SD)	50.2 ± 33.8	50.4 ± 33.7	49.4 ± 34.1	0.811	50.3 ± 32.5	50.0 ± 37.3	0.973
ALT (U/L, Mean ± SD)	29.8 ± 24.2	30.2 ± 24.8	28.7 ± 22.3	0.436	30.1 ± 24.4	29.1 ± 23.7	0.613

Values are expressed as *n* (%) or Mean ± SD. WBC, white blood cells; Alb, albumin; TBIL, total bilirubin; DBIL, direct bilirubin; AST, aspartate transaminase; ALT, alanine transaminase; SD, standard deviation.

Based on the disparate distribution of baseline characteristics between two groups.

The propensity score matching was performed using the fuzzy matching method, where nearest-neighbor matching was conducted within a caliper width of 0.02 based on the propensity scores (PS) and additional covariates, including age, gender, weight, pulmonitis, WBC, Hb, creatinine, Alb, Na+, K+, TBIL, DBIL, AST and ALT. Finally, 314 and 316 patients were included in the pre NT-proBNP PS-matched cohort and post NT-proBNP 24-h PS-matched cohort, respectively. There wasn't significant difference of baseline characteristics between low and high NT pro-BNP groups in PS-matched cohorts (Table 2).

(1) Short-term clinical outcomes

**Table 2:** Patient characteristics of low and high NT pro-BNP groups in PS-matched cohorts.

	Pre NT-proBNP PS-matched cohort (*n* = 314)			Post-24 h NT-proBNP PS-matched cohort (*n* = 316)		
	Low	High	*P*	Low	High	*P*
Demographics						
Age (year, Mean ± SD)	2.0 ± 0.8	2.0 ± 0.8	0.559	2.0 ± 0.8	1.9 ± 0.8	0.279
Gender (%)			0.818			0.649
Male	93 (59.2%)	95 (60.5%)		89 (56.3%)	93 (58.9%)	
Female	64 (40.8%)	62 (39.5%)		69 (43.7%)	65 (41.1%)	
Weight (kg, Mean ± SD)	4.6 ± 1.0	4.7 ± 1.1	0.765	4.7 ± 1.1	4.7 ± 1.1	0.986
Comorbidities and complications						
Pulmonitis (%)	36 (22.4%)	31 (19.9%)	0.491	29 (18.4%)	23 (14.6%)	0.362
Trisomy 21 syndrome (%)	5 (1.3%)	3 (2.5%)	0.720	6 (3.8%)	2 (1.3%)	0.283
Laboratory on admission						
WBC (mcL, Mean ± SD)	9.5 ± 4.3	9.4 ± 4.2	0.857	9.9 ± 4.1	9.7 ± 4.8	0.774
Hemoglobin (g/dl,Mean ± SD)	102.9 ± 14.5	102.6 ± 13.2	0.827	103.3 ± 14.1	103.1 ± 13.2	0.906
Creatinine (mg/dl, Mean ± SD)	21.7 ± 5.7	22.0 ± 5.4	0.584	21.6 ± 5.2	21.5 ± 5.7	0.758
Alb (g/L, Mean ± SD)	40.0 ± 3.9	40.2 ± 3.9	0.527	40.2 ± 4.1	40.4 ± 3.7	0.734
Na (mmol/L, Mean ± SD)	137.1 ± 3.0	136.8 ± 3.1	0.365	137.5 ± 3.3	137.2 ± 2.7	0.327
K (mmol/L, Mean ± SD)	4.3 ± 0.5	4.3 ± 0.7	0.673	4.3 ± 0.6	4.3 ± 0.7	0.757
TBIL (mg/dl, Mean ± SD)	21.0 ± 30.0	20.9 ± 31.3	0.982	20.7 ± 32.2	20.8 ± 32.0	0.970
DBIL (mg/dl, Mean ± SD)	6.1 ± 5.2	5.7 ± 4.2	0.488	6.0 ± 5.3	5.8 ± 4.4	0.720
AST (U/L, Mean ± SD)	49.2 ± 37.4	49.8 ± 33.2	0.890	51.6 ± 37.8	50.0 ± 37.7	0.709
ALT (U/L, Mean ± SD)	27.9 ± 17.3	28.8 ± 18.5	0.643	30.1 ± 24.4	29.1 ± 23.7	0.725

Values are expressed as *n* (%) or Mean ± SD. PS, propensity score; WBC, white blood cells; Alb, albumin; TBIL, total bilirubin; DBIL, direct bilirubin; AST, aspartate transaminase; ALT, alanine transaminase; SD, standard deviation.

In the pre NT-proBNP PS-matched cohort, compared with low NT pro-BNP group, the hospitalization time were longer in high NT pro-BNP group (*P* < 0.05). There wasn't significant difference of mechanical ventilation time, CPB time, CCU stay between the two groups (all *P* > 0.05). Correlation analysis was performed on pre NT-proBNP level and hospitalization time, and a positive correlation was demonstrated between the two variables (r = 0.16, *P* < 0.01) (Table 3). Meanwhile, in the post-op 24-h NT pro-BNP PS-matched cohort, there wasn't significant difference of mechanical ventilation time, CPB time, ACC time, CCU stay, and hospitalization time between low and high NT pro-BNP groups (all *P* > 0.05) (Table 3).

(1) Mid-term clinical outcomes

**Table 3:** Short-term clinical outcomes of low and high NT pro-BNP groups in PS-matched cohorts.

	Pre NT-proBNP PS-matched cohort (*n* = 314)			Post-24 h NT-proBNP PS-matched cohort (*n* = 316)		
	Low	High	*p*-value	Low	High	*p*-value
Mechanical ventilation time (h)	36.9 ± 36.2	43.9 ± 44.1	0.160	39.9 ± 44.5	40.3 ± 32.6	0.370
CPB time (min)	66.3 ± 26.8	69.8 ± 21.3	0.182	64.7 ± 23.7	69.9 ± 26.3	0.099
ACC time (min)	37.3 ± 13.9	40.4 ± 14.9	0.174	37.5 ± 14.3	39.5 ± 15.7	0.531
CCU stay(day)	4.4 ± 4.1	5.3 ± 4.4	0.066	4.5 ± 4.5	4.7 ± 3.6	0.140
Hospitalization time (day)	13.1 ± 1.8	14.8 ± 6.7	0.006	13.3 ± 5.0	14.0 ± 5.7	0.406

Values are expressed as Mean ± SD. PS, propensity score; CPB, Cardiopulmonary bypass; ACC, aorta cross-clamp; CCU, Intensive care unit.

**Figure 2:**
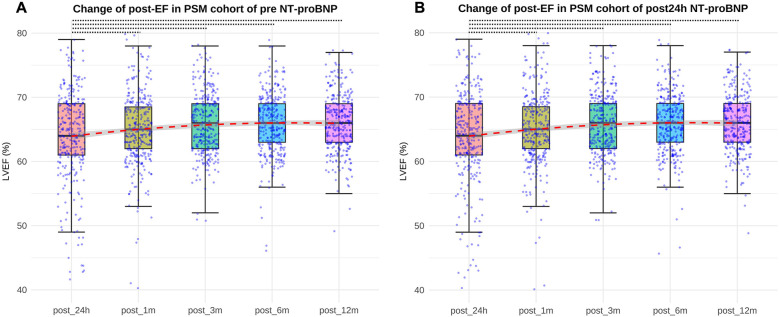
Differences between post-op 24-h EF and post-op 3-month, 6-month and 12-month EF both in pre-op and post NT-proBNP PS-matched cohort (all *P* < 0.05, represented by the black dashed line). **(A)** EF values at post-op 24 h, 1month, 3 months, 6 months, 12 months in the pre NT-proBNP PS-matched cohort; **(B)** EF values at post-op 24 h, 1month, 3 months, 6 months, 12 months in the post24 h NT-proBNP PS-matched cohort. EF, ejection fraction. pre-op, preoperative. post-op, postoperative.

In the pre NT-proBNP PS-matched cohort, the EF values at post-op 24-h, 1-month, 3-month, 6-month and 12-month were 65% (61%,69%), 65% (61%,69%), 65% (63%,69%), 66% (63%,69%), and 66% (63%,69%), respectively. In the post-op 24-h NT pro-BNP PS-matched cohort, the EF values were 64% (60%,69%), 65% (61%,69%), 66% (62%,69%), 66% (63%,69%), 66% (63%,69%) in the corresponding time points. Both in the pre-op and post-op 24-h NT pro-BNP PS-matched cohorts, there wasn't significant difference between post-op 24-h EF and post-op 1-month EF(*P* > 0.05), but there were significant differences between post-op 24-h EF and post-op 3-month, 6-month and 12-month EF, respectively (all *P* < 0.05) (Figure 2).

In the pre NT-proBNP PS-matched cohort, the post-op 1-month EF value was significant higher in low NT pro-BNP group compared with high NT pro-BNP group (*P* < 0.01). There wasn't significant difference of post-op 24-h, 3-month, 6-month and 12-month EF values between the two groups. In the post-op 24-h NT pro-BNP PS-matched cohort, there wasn't significant difference of post-op 24-h, 1-month, 3-month, 6-month and 12-month EF values between the two groups (Table 4).

**Table 4:** Ef values of low and high NT pro-BNP groups in PS-matched cohorts.

EF (%)	Pre NT-proBNP PS-matched cohort (*n* = 314)			Post NT-proBNP PS-matched cohort (*n* = 316)		
	Low	High	*p*-value	Low BNP	High BNP	*p*-value
Post-op 24h	64 (61,68)	60 (64,69)	0.962	65 (61,69)	64 (60,69)	0.939
Post-op 1m	65 (63,69)	64 (61,68)	0.004	65 (61,68)	65 (62,69)	0.904
Post-op 3m	66 (62,70)	65 (62,69)	0.273	66 (63,69)	65 (62,69)	0.181
Post-op 6m	66 (63,70)	65 (62,69)	0.072	66 (63,69)	65 (62,70)	0.980
Post-op 12m	66 (63,69)	66 (63,68)	0.300	66 (63,69)	66(62,70)	0.650

Values are expressed as median (25th–75th percentile). PS, propensity score; EF, ejection fraction; Pre-op, preoperative; Post-op, postoperative.

## Discussion

NT pro-BNP has been extensively studied in adult heart diseases as a cardiac neurohormone (7–11). It is significantly elevated in adult congestive heart failure patients and correlates with the severity of heart failure and predicts morbidity and mortality (8, 9). This indicator has also garnered attention in the field of congenital heart disease, with several surgical cohort studies reporting that NT-proBNP is associated with a higher risk of prolonged ICU stay after surgery (10, 11) and could enhance postoperative risk prediction models for congenital heart disease (12). However, these findings have not been consistently validated across all cohorts (13, 14), leaving the role of NT-proBNP in postoperative outcomes for congenital heart disease subject to debate.

Previous studies had reported no significant correlation existed between plasma BNP level and left ventricular EF in patients with acute Kawasaki disease (15). In the cohort of children in the emergency department, BNP was significantly elevated in patients with congenital or acquired heart disease (16). In patients with left-to-right shunts, BNP was elevated and positively correlated with shunt volume, systolic right ventricular pressure, and pulmonary resistance (17). There was no correlation between BNP and degree of ventricular hypertrophy (17). There was no significant increase in BNP in patients with tetralogy of Fallot (17). Plasma BNP levels were elevated in patients with functional single ventricle hearts and were not reduced after volume unloading by cavopulmonary connection (17). The above studies suggested that plasma BNP may have a variety of manifestations in children with diverse heart diseases.

The study of BNP in VSD is still limited. Previous study has reported BNP reflected pressure and volume loads and may help to identify children with VSD complicated by pulmonary hypertension (18). BNP levels were significantly linearly correlated with VSD defect size and positively correlated with pulmonary-systemic vascular flow ratio (Q_P_/Q_S_) (19). However, for children with VSD repair surgery, whether BNP is associated with postoperative clinical outcomes has not been studied. VSD is a congenital disease with a high incidence, and most of them require surgical repair (20). BNP measurement is a routine item in clinical practice. Identifying whether BNP has a predictive value on children with VSD surgery can provide reference on clinical treatment.

Both c-terminal BNP 32 and NT-proBNP are decomposition products of BNP precursors. NT-proBNP is a stable protein without any physiological activity. It has longer half-life period (about 70 min to 5 min) than c-terminal BNP 32 and has higher level (2–10 times) in patients with cardiac insufficiency (21, 22). Therefore, our research chose NT-proBNP as indicator for the sensitive and stable characteristics. In our study, NT-proBNP was measured preoperatively and 24-h postoperatively. NT-proBNP detections at these two time points were routine procedures in clinical work, and we were very interested in knowing whether this routine detection item had predictive value on the prognosis of VSD.

In the overall cohort, NT-proBNP levels did not differ between genders. NT-proBNP levels showed a gradual decline with age, which is consistent with previous findings (23). The NT-proBNP level was significantly higher at 24 h after surgery than before surgery, which may be due to the congestive heart failure caused by volume overload after surgery (24). Paul's study showed that the postoperative BNP level was significantly lower than that before the operation (25). This is because the BNP level was measured at 4–10 months after the operation in their study, when the volume load and pressure load of the heart had gradually returned to be normal, and secretion of NT-proBNP from ventricular myocytes decreased. In our overall patient cohort, the high NT-proBNP group had a younger age, lower body weight, higher incidence of pneumonia, and higher level of creatinine, TBIL, and DBIL. The different distribution of these baseline data may be since plasma BNP level in children with VSD correlate with Q_P_/Q_S_, mean pulmonary arterial pressure, and pulmonary-systemic vascular resistance ratio (18, 26), and the concomitant increased venous pressure could reduce renal blood flow and urine flow, resulting in elevation of creatinine, TBIL, and DBIL (27, 28). Differences in distribution of baseline characteristics data may lead to confounding effects on the clinical outcomes, so we used PS to obtain comparable cohorts.

In the pre NT-proBNP PS-matched cohort, the high BNP group had longer hospital stay, but there wasn't significant difference of mechanical ventilation time, CPB time, ACC time, and CCU stay between high NT pro-BNP group and low NT pro-BNP group. The results indicated that VSD patients with high pre-op BNP level had longer hospital stay than those with low pre-op BNP level. This could be caused by that pre-op BNP secretion was a response to ventricular volume and pressure load. Higher pre NT-proBNP level presented heavier ventricular burden and pronouncer hemodynamic changes, which would require longer treatment time to recover after surgery. In the post-op 24-h NT pro-BNP PS-matched cohort, there wasn't significant difference of short-term clinical outcomes between high and low NT pro-BNP group. This may be due to the increased post-op 24-h NT-proBNP was a transient change response to congestive heart failure caused by volume overload, which correlated with the VSD disease itself hardly and therefore didn't have relationship with short-term clinical outcomes.

Both in pre-op and post-op 24-h NT pro-BNP PS-matched cohorts, there wasn't significant difference between post-op 24-h EF value and post-op 1-mont EF value, but there were significant differences between post-op 24-h EF value and post-op 3-month, 6-month, 12-month EF values. This indicted that the cardiac function did not change significantly from 24 h to 1 month after operation, and a period of 3 months may be required for cardiac function improvement for children with VSD surgery. In addition, in pre NT-proBNP PS-matched cohort, the EF value at 1 month after surgery was higher in low NT pro-BNP group compared with the high group, and EF values from 3 months to 12 months postoperatively did not differ between the two groups. It indicated that VSD patients with high pre NT-proBNP level had worse cardiac function than patients with lower level at 1 month after surgery, but then acquired equal cardiac function improvement subsequently from 3 months after surgery. In the post-op 24-h NT pro-BNP PS-matched cohort, there was no difference in EF values between the two groups from 24 h to 12 months after surgery, which further illustrated that post-op 24-h NT-proBNP change was a transient response, and didn't have relationship with mid-term clinical outcome for children with VSD surgery. Therefore, in clinical practice, the necessity of measuring NT pro-BNP 24 h postoperatively as a routine item in VSD patients may be debatable, since from our findings it didn't have any relevance to short- and mid-term outcomes of the disease.

### Study limitations

There are still some limitations in our study. Firstly, the patient cohort we included was 0–4 years old, which did not include all age groups and older patients may have different disease characteristics and distinct outcomes. Secondly, the clinical symptoms of VSD patients have been significantly improved after surgery, and personal description of clinical symptoms is subjective. We adopt ultrasonic indicator EF value to evaluate the mid-term clinical outcome because EF continues to be a mature, stable, objective, recognized indicator of cardiac function despite some limitations (29). Recently, an increasing number of sensitive ultrasound indicators have gradually appeared, which can better reflect the recovery of cardiac function. In future studies, more updated and sensitive ultrasound indicators can be added to evaluate the cardiac function.

## Conclusions

VSD children with higher pre NT-proBNP level had longer hospital stays after surgery than those with lower level. Pre NT-proBNP level had no effect on mechanical ventilation time, CPB time, ACC time and CCU stay and cardiac function after 3 months postoperatively. Post-op 24-h NT pro-BNP level was not associated with short-term and mid-term clinical outcomes.

## Data Availability

The raw data supporting the conclusions of this article will be made available by the corresponding author upon reasonable request.
